# Carbon-Based Textile Sensors for Physiological-Signal Monitoring

**DOI:** 10.3390/ma16113932

**Published:** 2023-05-24

**Authors:** Wancheng Shao, Tianrui Cui, Ding Li, Jinming Jian, Zhen Li, Shourui Ji, Aobo Cheng, Xinyue Li, Kaiyin Liu, Houfang Liu, Yi Yang, Tianling Ren

**Affiliations:** 1 School of Integrated Circuit, Tsinghua University, Beijing 100084, China; 2 Beijing National Research Center for Information Science and Technology (BNRist), Tsinghua University, Beijing 100084, China; 3 Center for Flexible Electronics Technology, Tsinghua University, Beijing 100084, China

**Keywords:** carbon-based materials, textile sensors, physiological-signal monitoring

## Abstract

As the focus on physical health increases, the market demand for flexible wearable sensors increases. Textiles combined with sensitive materials and electronic circuits can form flexible, breathable high-performance sensors for physiological-signal monitoring. Carbon-based materials such as graphene, carbon nanotubes (CNTs), and carbon black (CB) have been widely utilized in the development of flexible wearable sensors due to their high electrical conductivity, low toxicity, low mass density, and easy functionalization. This review provides an overview of recent advancements in carbon-based flexible textile sensors, highlighting the development, properties, and applications of graphene, CNTs, and CB for flexible textile sensors. The physiological signals that can be monitored by carbon-based textile sensors include electrocardiogram (ECG), human body movement, pulse and respiration, body temperature, and tactile perception. We categorize and describe carbon-based textile sensors based on the physiological signals they monitor. Finally, we discuss the current challenges associated with carbon-based textile sensors and explore the future direction of textile sensors for monitoring physiological signals.

## 1. Introduction

With the development of society, the advancement of science and technology, and the arrival of the aging society, there is a growing concern for personal health [1]. In recent years, there has been a rising demand for wearable sensors. Numerous types of research on sensors for physiological-signal monitoring have been reported, including motion of the body and muscles [2], heart rate (HR) [3], SpO_2_, body temperature [4], pulse, respiration, respiratory humidity [5], and electrophysiological signals (electrocardiogram (ECG), electromyogram (EMG), electrooculogram (EOG), etc.) [6]. Sensors in wearable devices need to withstand mechanical deformation and enable measurements on non-flat surfaces. Traditional sensors cannot meet the application needs because of their rigidity [7]. Nowadays, research focuses on flexible wearable sensors that can attach comfortably to the human body, accurately monitor physiological signals, and facilitate long-term monitoring. Moreover, these sensors are becoming increasingly compact and integrated [8].

At the same time, various types of comfortable textiles are commonly used in daily life. By combining sensitive materials and electronic circuits with textile, flexible textile sensors can be manufactured to monitor long-term physiological signals, which can achieve high comfort and durability while maintaining high performance with high comfort and durability [9]. 

**Figure 1:**
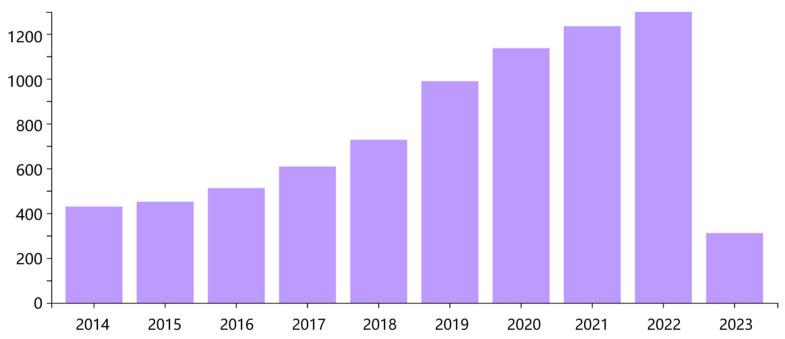
A year-by-year breakdown of the total number of publications under keywords sensor and fabric/textile. Source: Web of Science database, May 2023.

The main international database for the literature search in this review was the Web of Science. The search keywords for topics and titles included (1) “sensor” and (2) “textile” or “fabric”. The types of articles in this literature search include research papers, review papers, and conference papers. The articles were published in the last 10 years, from 2014 to 2023. The literature search returned 7695 references. Figure 1 clearly shows the number of articles published in the textile sensor field over the past 10 years. Then, the found literature was further screened, and the screening criteria were as follows: (1) written in English; (2) peer-reviewed journal articles, excluding official governmental reports; (3) carbon-based materials, including graphene, CNTs, and CB, used in the fabrication of flexible sensors; and (4) focused on the application of human physiological-signal monitoring. This left 1539 studies. Finally, we classified them according to the different carbon-based materials and physiological signals and selected 85 typical articles as references. It can be seen that there is an increasing number of research on flexible textile sensors. In addition, with the growth of market demand, this field will certainly have important research value and development potential in the future.

Currently, the main way to manufacture textile sensors is to attach the conductive material to the surface of the textile or to weave the sensing yarn directly [10]. Some common textile materials used as substrates such as cotton, nylon, silk, polyurethane (PU), and polyvinylidene fluoride (PVDF) can be applied to textile sensors, which make sensors flexible and breathable. Several textile production methods such as embroidery, sewing, knitting, weaving, and coating have also been used in the manufacturing of textile sensors [11]. Conventional physiological-signal-monitoring sensors are unsuitable for long-term wearing because of their rigid structure, and ordinary flexible sensors are not natural when worn. However, textile sensors can avoid these problems. Textile sensors have a similar appearance to the traditional textile, which is easier for people to accept psychologically without social stigma and psychological pressure. Another advantage is that flexible textile sensors are more convenient for daily use. Such sensors are easier for users to wear in daily life and more effective at monitoring physiological signals. For example, it is an important process to determine whether a joint or ligament is injured or damaged, but these locations are often difficult to measure. The flexibility and stretchability of the textile sensors allow measurement directly on the patient’s joint [12,13]. Therefore, textile sensors with non-invasive monitoring and decent appearance are bound to be an important research direction in the field of wearable devices.

There are many sensitive materials for making textile sensors. Researchers have studied conductive polymers such as polypyrrole and PEDOT to create conductive textiles [14,15]. In addition, researchers also use carbon-based materials to create flexible textile sensors. Carbon-based materials such as graphene, carbon nanotube (CNT), and carbon black (CB) have been widely used in textile electronics due to their relatively low mass density, high flexibility, and high electrical conductivity [10]. Most flexible textiles are not electrically conductive. Carbon materials attached to the surface of textiles can form conductive coatings, which can be used in the design and manufacture of various textile sensors. 

Recently, several reviews have been reported of wearable flexible sensors for physiological-signal monitoring [16,17,18]. However, most reviews focus on the development of carbon-based strain sensors for physiological-signal monitoring [16,17]. A detailed overview of wearable flexible sensors combining carbon-based materials and textiles for physiological-signal monitoring has not been reported. In this review, we focus on the latest development of carbon-based textile sensors for physiological-signal monitoring and systematically summarize the design methods, preparation processes, and performances of carbon-based textile sensors. The first part introduces some carbon-based materials used in textile sensors. This part describes the development and characteristics of graphene, CNT, and CB, respectively, as well as some application examples in the field of flexible textile sensors. The second part discusses different types of carbon-based textile sensors for monitoring different physiological signals, including ECG, human body movement, pulse and respiration, body temperature, and tactile perception. Finally, we present a perspective on the current challenges and future development of carbon-based textile sensors in practical application. We suggest possible development directions of textile sensors for physiological-signal monitoring in the future in terms of smart textile sensing systems and list some existing issues that need to be addressed in the development of textile sensors.

## 2. Carbon-Based Materials Used in Textile Sensors

To make flexible electronic devices, either deformable structures are designed, or flexible stretchable materials are used [19]. It is also worth noting that electrical circuits generate heat during operation, and electronic textiles are no exception. Typical textile materials have poor thermal conductivity, and the human body can withstand a limited range of temperatures, so the inclusion of highly thermally conductive materials should be considered in the manufacturing of electronic textile materials to facilitate the rapid release of heat generated by the circuit [20]. Most carbon-based materials have excellent thermal conductivity. For example, the thermal conductivity of graphene can even reach 5000 W/(K∙m) [21]. Moreover, carbon-based materials have advantages such as good electrical conductivity, low toxicity, low mass density, and easy functionalization. Therefore, carbon-based materials have great application potential in the field of wearable electronic devices. The following are the properties and characteristics of carbon-based materials commonly used in flexible sensors. 

### 2.1. Graphene

**Figure 2:**
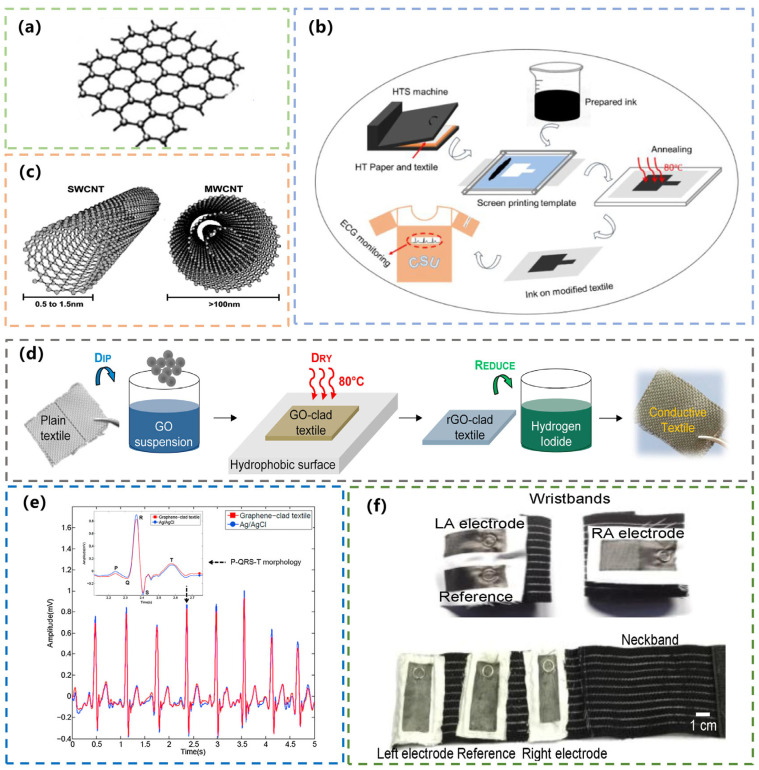
(**a**) Basic structure of graphene. Reprinted with permission from Ref. [26]. Copyright 2021, MDPI. (**b**) The preparation process of graphene-coated textile electrodes. Reprinted with permission from Ref. [27]. Copyright 2020, IOP Publishing Ltd. (**c**) Schematic diagram of SWNT and MWNT. Reprinted with permission from Ref. [30]. Copyright 2017, ASSOC BRASIL POLIMEROS. (**d**) Diagram of synthesizing graphene-coated textiles. Reprinted with permission from Ref. [31]. Copyright 2015, ELSEVIER SCIENCE SA. (**e**) Graphene-clad textiles for ECG monitoring. Reprinted with permission from Ref. [31]. Copyright 2015, ELSEVIER SCIENCE SA. (**f**) The integration of graphene-clad textiles into elastic bands. Reprinted with permission from Ref. [32]. Copyright 2017, MDPI.

Graphene is a two-dimensional carbon nanomaterial consisting of carbon atoms with sp² hybrid orbitals and a hexagonal honeycomb lattice. It was first isolated from graphite in 2004, and its discovery was significant. It is only a few atoms thick and has excellent stability, conductivity, and mobility of up to 10,000 square centimeters per volt-second at room temperature [22]. Since its inception, graphene has been studied in different directions and applied in different fields. Graphene has excellent electrical conductivity, thermal conductivity, optical transparency, and flexibility. In the field of carbon-based flexible wearable sensors, it is also an important material, such as in pressure sensors, temperature sensors, biosensors, strain sensors, etc., and it is further applied to the monitoring of human vital sign characteristics [23]. The preparation of graphene for wearables typically involves two methods. One is the growth of graphene by chemical vapor deposition (CVD) [24]. In the other, graphene is extracted by exfoliation [25]. Compared with the CVD method, natural graphite-stripped graphene has the advantages of mass production and low cost, which are conducive to practical applications [19]. The basic structure of graphene is shown in Figure 2a [26].

Now, there have been many applications of graphene materials in textile sensors for monitoring human physiological signals. In 2019, Xu et al. developed a graphene-coated textile electrode for ECG monitoring. The graphene electrodes were prepared by screen printing on a modified textile substrate using graphene ink (Figure 2b). The resistance of the electrode can be changed by controlling the number of prints. The graphene textile electrode has good washable resistance and flexibility. The performance of ECG signals obtained by this electrode is comparable to that of conventional Ag/AgCl electrodes. In addition, the ECG signal can still maintain good performance after repeated washing and bending [27]. In 2022, Eom et al. also researched and developed a graphene-based piezoresistive pressure sensor. An electrically conductive textile was formed using a dispersed solution of graphene and polyurethane. The developed sensor meets the requirements of flexibility, tension, and air permeability. It can be used to monitor human movement and pulse signals [28]. In recent years, researchers have been exploring the use of graphene in flexible sensors. At present, studies on graphene derivatives such as graphene oxide (GO), reduced graphene oxide (rGO), and graphene nanoplatelets (GNPs) in the field of flexible sensors have also been reported [29]. In the future, graphene and its derivatives will certainly have wider applications in textile sensors.

### 2.2. CNT

CNT, a quasi-hollow tubular nanomaterial made of carbon atoms, was discovered in 1991 by Dr. Lijima in Japan [33]. CNTs can be divided into single-walled nanotubes (SWNTs) and multi-walled nanotubes (MWNTs) (Figure 2c). SWNTs are made of a single layer of graphene wound in a certain direction, while MWNTs are made of different direct SWNTs nested coaxially. CNTs have excellent electrical and thermal conductivity, with an electrical conductivity of up to 10^−7^ S/m and a thermal conductivity of up to 3500 W/(K∙m). At the same time, they have good tensile strength, with an elongation at a break of 15%–30%. Even under stretching or bending conditions, the original electrical properties can be guaranteed to remain unchanged [30,34].

There are also some reports about the application of CNTs in carbon-based textile sensors for human physiological-signal monitoring. In 2011, a conductive yarn prepared by mixing polylactic acid (PLA) with 4 wt% CNTs was reported to be used as a humidity textile sensor [35]. However, this was only applicable to environmental humidity measurement. With the developments of recent years, more physiological-signal sensors have been studied. In 2019, Bansal et al. reviewed the development of electrodes based on CNTs for long-term and continuous ECG monitoring, which can help monitor the health of the human body [36]. In 2023, Hossain et al. used 3D knitted technology to integrate sensing yarns wrapped in CNTs into textiles. The textile structures can accurately monitor human movement and maintain the same properties through 30 washing cycles [37].

### 2.3. CB

In addition to these two new carbon materials mentioned above, CB, an old material, has also been used in the manufacture of textile sensors. CB has high conductivity and high surface area and is relatively cheap [38]. Some related studies have been reported. In 2016, Melnykowycz et al. produced a strain sensor based on a mixture of CB and thermoplastic elastomer (TPE). The sensor can be used to measure human movement and monitor pulse waves by attaching it to the elastic band of the textile. The CB/TPE structure enables the sensor to have good stretchability, good adhesion to the human skin surface, and sensitivity to the movement of the body [39].

## 3. Carbon-Based Textile Sensors for Different Physiological-Signal Monitoring

In the field of flexible wearable sensors for human physiological-signal monitoring, the main sensors involved include ECG electrodes/sensors, sensors for body motion monitoring, body temperature sensors, body humidity sensors, tactile sensors, etc. [40]. To stick to the human body for long-term use, these textile sensors need to be lightweight, breathable, flexible, stretchable, and washable [41,42]. There has been a lot of research on these textile sensors for monitoring different physiological signals.

### 3.1. Carbon-Based ECG Textile Electrodes/Sensors

Cardiovascular disease has become the highest cause of death with the advent of the aging society. Long-term ECG signals can help identify and treat potential cardiovascular disease risks. Therefore, it is important to develop ECG electrodes/sensors with high performance and long-term application [43]. Conventional Ag/AgCl ECG electrodes, also known as “wet” electrodes, use a gel to reduce their resistance to contact with human skin. However, in some cases, skin irritation and allergic reactions have been reported due to the long-term use of gel “wet” electrodes. In addition, Ag/AgCl electrode performance declines over time due to the drying of the gel [44]. Therefore, conventional Ag/AgCl electrodes are not conducive to long-term monitoring applications. One solution is to make dry electrodes from conductive textiles. These electrodes are highly comfortable and suitable for long-term monitoring applications [45].

#### 3.1.1. Graphene-Based ECG Textile Electrodes/Sensors

Graphene with high conductivity is used in the study of ECG textile electrodes and sensors. In 2015, Yapici et al. proposed a GO-wrapped textile electrode for ECG monitoring. The first step was to prepare GO suspension. A total of 2 g of graphite powder (particle size less than 20 μm) and 7 g of potassium permanganate was added into 50 mL concentrated sulfuric acid, and then distilled water was added and stirred in. GO was stripped off through centrifugation and sonication. The GO suspension was obtained by hydrochloric acid treatment and distilled water washing. Finally, the nylon textile with a hydrophilic surface was immersed in it and then placed on a hydrophobic carrier. After drying, the conductive nylon was evenly wrapped with graphene. The GO textile electrode was prepared, and its preparation process is shown in Figure 2d. Before and after coating GO, the conductivity of nylon changed from 6 × 10^−12^ S/cm to 4.5 S/cm. There was a huge increase in conductivity. Nylon has a higher conductivity when coated with GO because of its low surface roughness [46]. Compared to conventional Ag/AgCl electrodes, the GO electrode shows better performance in ECG measurements with higher fidelity to the signal (Figure 2e). In addition, it even could be used for EMG or EEG monitoring, as in the following study [31]. In 2017, Yapici et al. improved the performance based on the above and investigated possible applications of dry electrodes. A piece of nylon textile was immersed in a GO suspension and heated to form a conformal coating of GO sheets on the textile. Finally immersed in hydrogen iodide (HI), the GO was reduced and converted to graphene to form a highly conductive graphene-coated textile. The prepared sample was cut into a small size of about 6 cm × 3 cm to form the ECG electrode. It was then glued to a piece of cotton cloth, which allowed the electrodes to be easily attached to various types of clothing by sewing. Figure 2f shows some of its possible applications [32].

**Figure 3:**
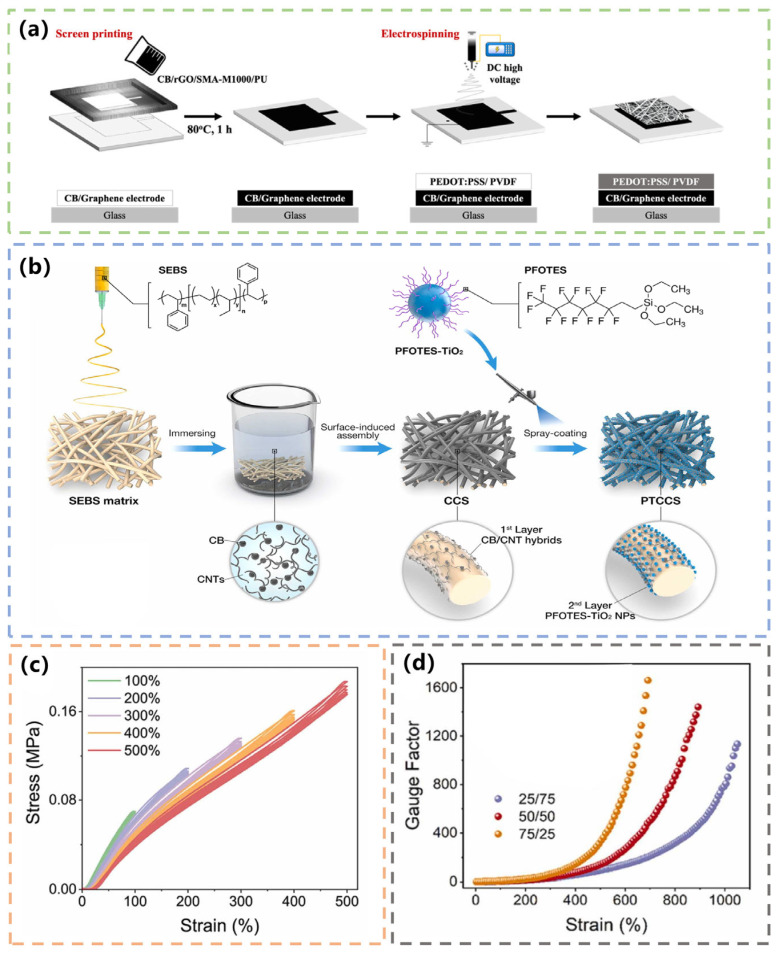
(**a**) Schematic diagram of the synthesis process for nanofiber carbon electrode. Reprinted with permission from Ref. [49]. Copyright 2021, AMER CHEMICAL SOC. (**b**) The process of the ultra-stretchable and superhydrophobic textile-based bioelectrode. Reprinted with permission from Ref. [52]. Copyright 2022, ELSEVIER. (**c**) The strain–stress curves of the textile sensor with strains from 100–500% for five cycles. Reprinted with permission from Ref. [52]. Copyright 2022, ELSEVIER. (**d**) Plots of GF value versus strain of the strain sensor with different CB/CNT volume ratios. Reprinted with permission from Ref. [52]. Copyright 2022, ELSEVIER.

In addition to common clothing materials, textile sensors can also be made by combining some synthetic flexible polymer materials with carbon-based materials [47]. Research on the application of conductive nanofiber composites in ECG monitoring systems has been lacking. In 2021, Huang et al. developed a stretchable, flexible nanofiber-membrane carbon electrode that can be used for ECG monitoring. The carbon electrode was composed of rGO, CB, and PU. The nanofibers were made by mixing PVDF and poly (3, 4-ethylene dioxythiophene) polystyrene sulfonate (PEDOT/PSS) using electrospinning technology (Figure 3a). Electrospinning offered a fast and easy way to prepare nanofibers [48]. During electrostatic spinning, the polymer solution was charged at high pressure, causing the solvent to evaporate, and subsequently deposited on the collector as a fiber. The fibers prepared by electrospinning were nanoscale and had a high specific surface area. The nanofiber carbon electrode has high conductivity, water resistance, and durability, which is suitable for long-term physiological-signal monitoring [49].

#### 3.1.2. CNT/CB-Based Textile Electrodes/Sensors for ECG Monitoring

Flexible sensors based on CNTs generally show a large sensing range but low sensitivity or high sensitivity but a small sensing range [50,51]. To solve this problem, Dong et al. developed a synergistic CB–CNT-combination stretchable conductive network and superhydrophobic perfluorooctyltriethoxysilane-modified TiO_2_ nanoparticles (PFOTES-TiO_2_ NPs) in 2022. They designed and fabricated an ultra-stretchable self-cleaning non-woven textile bioelectrode with excellent health monitoring and antifouling performance. The preparation process involved dipping the Styrene-ethylene-butylene-styrene (SEBS) fabric spun by electrodeposition into a mixture of CNTs and CB, followed by brushing the mixture of PFOTES, ethanol, and TiO_2_ to form a superhydrophobic layer on the surface of the textile. Figure 3b shows the fabrication process of the bioelectrode. The SEBS film has high tensile properties with a stretching length of up to 1400%. SEBS is an ideal substrate for flexible sensors because it can maintain good resilience. Figure 3c shows the strain–stress curves of the textile sensor with a strain range of 100–500% for five cycles. The team used a mixture of CB and CNTs to balance the sensitivity and the sensing range. Figure 3d shows the GF-strain curves of the strain sensor with different CB/CNT volume ratios. In the strain range of 0–200%, the GF value of the sensor presents a linear distribution, and the sensitivity and sensing range can be adjusted by changing the CB content. Due to its low Young’s modulus and high sensitivity, it can be closely attached to the surface of the human body to detect the subtle deformation of physical activity. It can be used for ECG and myoelectric monitoring to obtain clear electrical signals [52].

### 3.2. Carbon-Based Textile Sensors for Body Motion Monitoring

In general, human motion signal monitoring can be divided into large movements such as joint and limb movements and small body deformations such as in the pulse and breathing [53]. Most carbon-based textile sensors that monitor human motion are flexible strain sensors. There has been a lot of research that focuses on the development of carbon-based strain sensors for physiological-signal monitoring. For more details, readers may consult the following recent reviews [16,17]. Strain sensors work by converting signals of mechanical deformation into electrical signals. According to different principles, they can be divided into resistance, capacitance, and piezoelectric sensors. The resistive sensor structure mainly consists of a conductive sensitive film and flexible substrate. The change in the conductive pathway caused by the strain leads to the corresponding change in resistance. The capacitive sensor structure is mainly composed of a flexible medium in the middle and conductive electrodes on either side of the medium. One main sensing mechanism of capacitive sensors is the deformation resulting in a change in the volume of the space between the electrodes and thus, a change in the capacitance of the sensor [16]. Piezoelectric sensors are mainly made of piezoelectric materials. The measurement principle is that stress causes lattice deformation of piezoelectric materials, which leads to the change of the crystal polar state. Therefore, as the piezoelectric material strains, it generates an internal electric field that converts pressure into electricity. Piezoelectric materials can be divided into four categories: ceramics, single crystals, polymers, and composites (composed of piezoelectric ceramics or single crystals in a polymer matrix) [54].

#### 3.2.1. Graphene-Based Textile Sensors for Body Motion Monitoring

**Figure 4:**
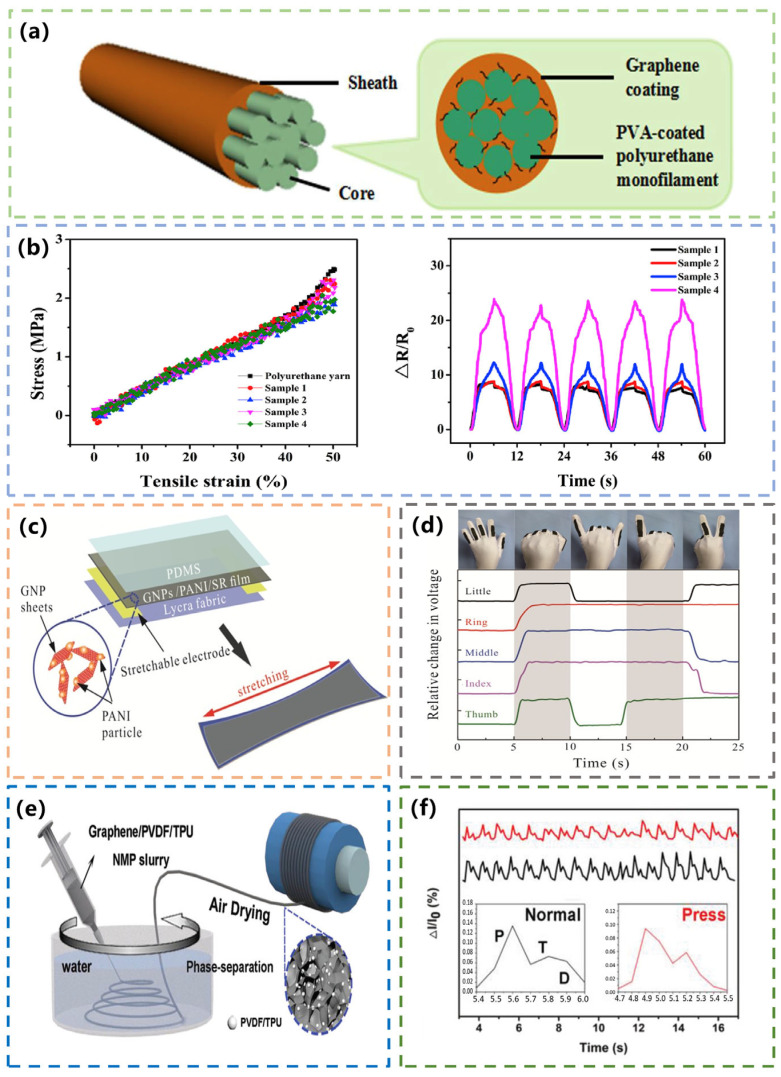
(**a**) Schematic structure of yarn sensor. Reprinted with permission from Ref. [59]. Copyright 2017, PERGAMON-ELSEVIER SCIENCE LTD. (**b**) The stress–strain relationship and the change in relative resistance of the four graphene/polyurethane-based sensors. Reprinted with permission from Ref. [59]. Copyright 2017, PERGAMON-ELSEVIER SCIENCE LTD. (**c**) Schematic structure of GNPs/PANI/SR strain sensor. Reprinted with permission from Ref. [60]. Copyright 2017, Wiley. (**d**) Relative voltage changes under different gestures. Reprinted with permission from Ref. [60]. Copyright 2017, Wiley. (**e**) Schematic process of PGFs. [61]. Copyright 2019, Wiley. (**f**) Wrist pulse resistance in the normal and pressed states. Reprinted with permission from Ref. [61]. Copyright 2019, Wiley.

Graphene is used to make strain sensors to monitor human movement. The combination of graphene with flexible textiles is also worth investigating. Yarns with sensing properties not only have a low cost and simple manufacturing process; they can use weaving technology to form a better sensing structure, and the measurement position is more flexible [55,56]. In 2017, Li et al. reported the preparation of a novel yarn strain sensor based on commonly used PU yarns that were easy to integrate into textile structures and apply textile technology to wearable applications (Figure 4a). The yarn was wrapped with a graphene/polyvinyl alcohol composite material as a conductive sheath and polyurethane yarn as an elastic core. A simple, scalable, and low-cost layered assembly method was adopted. By combining the advantages of graphene, such as superior electrical and mechanical properties and thermodynamic stability [57], with the advantages of polyurethane yarn, such as high tensile strength and elasticity, and good textile processing properties [58], a strain sensor with high sensitivity and good tensile strength was produced. Figure 4b shows the stress–strain relationship and the change in relative resistance of the four graphene/polyurethane-based sensors under a 50% stretch/release cycle. It can be seen that the sensor can almost maintain the good tensile strength and elasticity of its polyurethane core, including high tensile strength and linearity, as well as low hysteresis. When the strain is about 40%, the stress–strain curve of the sensor almost coincides with the stress–strain curve of the polyurethane core. When the strain exceeds 40%, the stress–strain curve of the sensor deviates only slightly from that of the core. This shows that coating the polyurethane core with graphene nanosheets does not significantly change the elasticity of the polyurethane yarn while providing high sensitivity [59].

Huang et al. applied a simple spin-coating technique to apply GNPs to textiles to make strain sensors and used polyaniline (PANI) particles in GNPs to improve sensor performance. In 2017, they prepared a textile strain sensor for human gesture recognition based on the highly conductive PANI polymer, GNPs, and silicon rubber (SR). As shown in Figure 4c, the structure of the textile strain sensor mainly includes the PDMS encapsulation layer, the conductive composite material (GNPs/PANI/SR), and the elastic PU fabric electrode. The resistance-sensitive material of the sensor is the GNPs/PANI/SR film. The addition of PANI particles improves the electrical conductivity of the thin film. The small-sized PANI particles are attached to the surface and edge of the GNPs. When the sensor is stretched, PANI particles fill the gaps generated by the stretched GNPs, not only maintaining the structure but also forming a collaborative conductive network with GNPs. This results in an increase in electrical conductivity. Five separate strain sensors were implanted into the spandex gloves to detect the bending and stretching of the fingers. An overview image of the data glove and the corresponding output waveform when the finger was bent and stretched are shown in Figure 4d. A rapid response of the signal can be observed during the bending process. The results show that the strain sensor can be used to monitor human motion [60].

To further improve the sensitivity of sensors, Huang et al. reported a polymer nanosphere decorated with graphene-composed porous fibers for wearable and highly sensitive strain sensors in 2019. Inspired by rolling friction, the group reduced the interconnections between graphene sheets and polymers by reducing the contact area. They designed porous graphene fibers (PGFs) with polymer nanospheres mixed with PVDF and PU inserted between the sheets (Figure 4e). This made the PGFs highly sensitive while being used for long durability. Pulse is a very important but relatively small physiological signal. This sensor can be used to monitor pulse waves, as shown in Figure 4f. The pulse signal was very clear with a beating frequency of 68 min^−1^. The insertion plot (black curve) showed a close-up of the peak of a single pulse and produced the typical characteristics of the pulse waveform, i.e., shock wave (P wave), tidal wave (T wave), and relaxation wave (D wave). When the vein was pressed, the signal strength (red curve) was lower than the normal signal and the pulse frequency dropped to about 60 min^−1^. This indicated the high sensitivity of PGFs to distinguish small changes [61].

#### 3.2.2. CNT-Based Textile Sensors for Body Motion Monitoring

**Figure 5:**
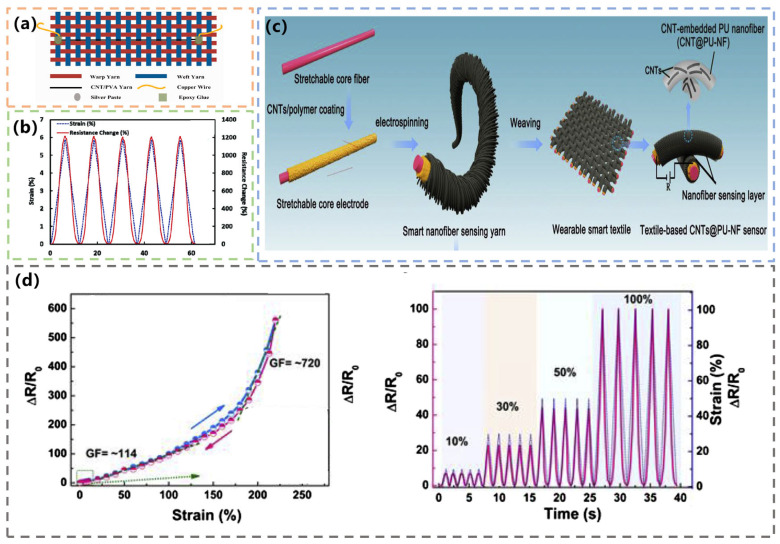
(**a**) Schematic of CNT/PVA yarn strain sensor. Reprinted with permission from Ref. [62]. Copyright 2018, SAGE PUBLICATIONS LTD. (**b**) Test of motion and the change of resistance. Reprinted with permission from Ref. [63]. Copyright 2022, IEEE. (**c**) Schematic process of CNTs@PU-NF sensor. Reprinted with permission from Ref. [64]. Copyright 2020, PERGAMON-ELSEVIER SCIENCE LTD. (**d**) Strain-sensing performance. Reprinted with permission from Ref. [64]. Copyright 2020, PERGAMON-ELSEVIER SCIENCE LTD.

CNTs are widely used in the manufacture of strain sensors because of their good electrical conductivity and stability. In 2018, Liu et al. proposed a new method to fabricate strain-sensing fabrics by embedding single-CNT strain-sensing yarns into textile structures. The yarns were coated with polyvinyl alcohol (PVA) to obtain higher mechanical properties (Figure 5a). The CNT-based yarns coated with PVA had better durability and mechanical properties. They performed with stable, repeatable, and linear sensing properties in the cyclic loading process. The CNT-based textile sensor showed a fast and accurate response to finger motion detection, demonstrating the potential of wearable electronics [62].

To improve the air permeability and comfort of fabric sensors, different methods have been used to make flexible sensors. In 2019, Doshi et al. used an innovative and scalable method of electrophoretic deposition to deposit CNTs onto knitted fabrics to develop a flexible, lightweight, and comfortable tensile sensor for human motion monitoring. Knitted fabrics consisted of 44% nylon, 43% polyester, and 13% spandex. Due to the chemical bonding of the CNTs to the fiber surface, the robustness of the coating ensured a repeatable response over multiple test cycles. Figure 5b shows the high sensitivity and stability of the textile strain sensor. When the sensor was tested at a strain of about 6% tension, the measured resistance change was about 1200% [63].

The high sensitivity and wide sensing range of strain sensors are usually contradictory. The strain sensors’ formation by the electrostatic spinning of sensing yarn can effectively improve their performance. In 2020, Qi et al. created a CNT-embedded PU nanofiber sensing yarn (CNTs@PU-NF). It was a stretchable, multimodal, wearable textile strain sensor made by adding CNTs to elastic PU nanofibers. First, stretchable, conductive, carbon-rich nanotube fibers were fabricated as core fibers and electrodes. CNTs were then added to elastic PU nanofibers and wrapped on stretchable core electrodes by one-step electrospinning to form stretchable piezoresistive nanofiber sensing yarns. Finally, these nanofiber sensing yarns served as warp and weft yarns that were perfectly integrated into the wearable textile substrate using weaving technology (Figure 5c). The sensor responded immediately to minute pressures with a fast response time of 30 ms. The sensors with nanofiber sensing layers of different thicknesses showed a monotone sharp increase in resistance with strain along different GF values and had a wide strain sensing range. The hysteresis of the sensors was negligible when the strain was applied back and forth between 0% and 40%, and only slight hysteresis was observed at tensile strains greater than 220%. The mean GF value of the sensors was 114 in the 100% strain range and 720 in the 100–220% strain range. Even at a tiny strain of 0–1%, they had a significant GF value of 54.9 (Figure 5d). The CNTs@PU-NF sensors showed high cyclic stability and reproducibility and can be used to monitor human daily movements [64].

#### 3.2.3. CB-Based Textile Sensors for Body Motion Monitoring

**Figure 6:**
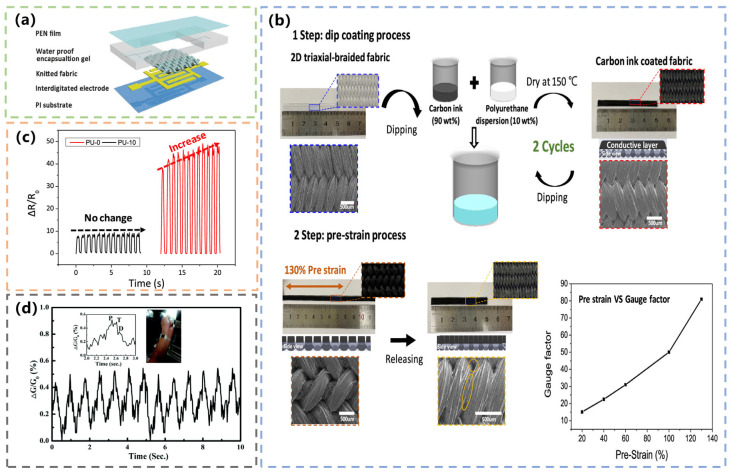
(**a**) Illustration of the sandwich structure of the textile sensor. Reprinted with permission from Ref. [66]. Copyright 2018, Wiley. (**b**) Dip-coating process and pre-strain process of sensor fabrication. Reprinted with permission from Ref. [67] Copyright 2021, American Chemical Society. (**c**) Performance of textile coated with PU. Reprinted with permission from Ref. [67] Copyright 2021, American Chemical Society. (**d**) Monitoring of pulse by the rGO/CNTs/NWF strain sensors. Reprinted with permission from Ref. [68]. Copyright 2020, ROYAL SOC CHEMISTRY.

CB is also used in the production of strain sensors because of its good electrical conductivity and low cost. Due to the inevitable inelastic deformation of the textile, response fluctuations and hysteresis still exist [65]. To address this limitation, Luo et al. created a flexible piezoresistive sensor (FPS) in 2018. CB particles and PVDF were added into knitted fabrics as electrical and mechanical interconnections between the fibers (Figure 6a). The knitted structure was interwoven yarn of twisted, conductive nylon fibers. The knitted fabric was decorated with CB grains. PVDF was introduced to improve the adhesion of CB to fibers. At the same time, it can form an interconnection between the fibers, thus filling the air gap and reducing hysteresis deformation. A CB/PVDF film uniformly covered the fiber surface and indeed formed interconnections between fibers in many areas. The sensor can capture subtle physiological-signal changes, such as deep inhalations and deep exhalations. The sensor can also be used to monitor superficial temporal artery pulse pressure and pulse wave velocity [66].

Although the method of coating CB directly on the flexible textile is simple and low-cost, it has low tensile strength and sensitivity. Therefore, it requires further research and exploration of diversified textile structures with higher performance. In 2021, Park et al. designed a strain sensor with high tensile strength and sensitivity using low-cost carbon-based ink. The carbon-based ink was mainly composed of CB, supplemented by gelatin and PU mixed, with characteristics of being waterproof, stable, and washable. Two-dimensional triaxial-braided weaving was used to obtain this textile, which was soaked in a carbon-based ink solution and dried so that enough CB was evenly attached to the surface of the textile. The initial resistance was measured at 20 kΩ. The carbon ink-coated textile was pre-strained 130% at a rate of 10 mm/min. Figure 6b shows the dip-coating and pre-strain process of macroscopic crack formation on the surface of carbon ink-coated textiles. In practical application, the cracks separate during tension and the resistance increases rapidly, thus enhancing the sensitivity of the sensor with GF up to 80. After the release of external force, the textile also returns to its original state. The existence of cracks also makes the sensor with air permeability suitable for long-term physiological-signal monitoring. The doping ratio of PU affects the adhesion between the carbon black and the fabric. Fabrics coated with a PU content of 10 wt% (PU-10) show excellent performance in the test (Figure 6c). The initial resistance remains unchanged before and after washing. It also shows high stability and long-term durability after 5000 cycles of tensile testing. After testing, the sensor can not only monitor the physiological information of low-scale movements such as breathing or pulse, but can also obtain the physiological information of large-scale movements such as joint movements [67].

#### 3.2.4. Graphene/CNT-Based Textile Sensors for Body Motion Monitoring

In addition to performance, the durability and washability of the sensors for daily use needs to be considered. CNTs have poor adhesion to textiles by coating or inkjet printing. In 2020, Tang et al. reported a machine-washable electronic textile sensor with high strain-resistance and high thermal conductivity. CNTs were fixed to non-woven fabric by the nano-soldering method, and then rGO was combined with the CNTs/NWF. The textile exhibited excellent properties. When the tensile strain was 1.0%, the strain coefficient was 32.65. It could detect subtle strains as low as 0.01%. The textiles showed excellent washability. After washing in a washing machine with a stirring rate of 700 rpm, the electrical resistance and thermal conductivity did not change much. At the same time, both rGO and CNTs were retained on the NWFs, and the carbon weight did not change significantly. The textile was used to measure human movement. For example, it was worn on fingers to record finger movements. The higher the curvature of the fingers, the greater the change in conductance is. When the finger returns to its original position, the conductance correspondingly returns to its original value. It can also be mounted on the hand to monitor pulse rate and can also record different human pulse waveforms (diastolic, tidal, and shock peaks) (Figure 6d) [68].

Nano-soldering methods can improve the adhesion of conductive materials on textile surfaces and can be also used in the fabrication of strain sensors. In 2021, Yao et al. reported highly sensitive gas-permeable strain sensors that are washable and wearable. The team created a sensor based on a mixture of graphene and CNTs on a non-woven fabric (NWF) substrate by using the ultrasonic nano-soldering method. The electronic textile had high electrical conductivity, was highly sensitive to tensile strain and pressure, and can be used as a wearable sensor for medical monitoring. The wearable rGO/CNTs/NWF sensors can monitor human movement by being worn on the index finger. When bending to different states, the sensor shows significant changes in resistance, and when the finger returns to the same position, the resistance values are nearly identical. In addition, the wearable rGO/CNTs/NWF sensors were used to detect pulse, muscle, and heart rate. They can record distinct human pulse waveforms (diastolic, tidal, and percussion peaks) at a pulse frequency of 12 per 10 s (72 beats/min), within the normal range for healthy adults. Significant pulse signals can be detected, and signal peaks are significantly dense. They can still maintain high electrical conductivity after machine washing. These results indicate that wearable rGO/CNTs/NWF sensors have excellent repeatability, washability, and durability and are sensitive to both weak physical stimuli and large movements; therefore, they have great potential applications in smart clothing, personal healthcare, human movement, and robotics [69].

To deal with the complicated manufacturing method of textile sensors, Zhou et al. reported a textile strain sensor based on screen printing technology to transfer graphene-nanosheets (GNSs)/NWCNT mixed ink to cloth tape. It has good linearity and stability with low manufacturing costs. The sensing range and sensitivity of the textile sensor can be adjusted according to the different design methods, which can be used in human motion monitoring such as gesture recognition [70].

### 3.3. Carbon-Based Humidity Textile Sensors for Respiration Monitoring

**Figure 7:**
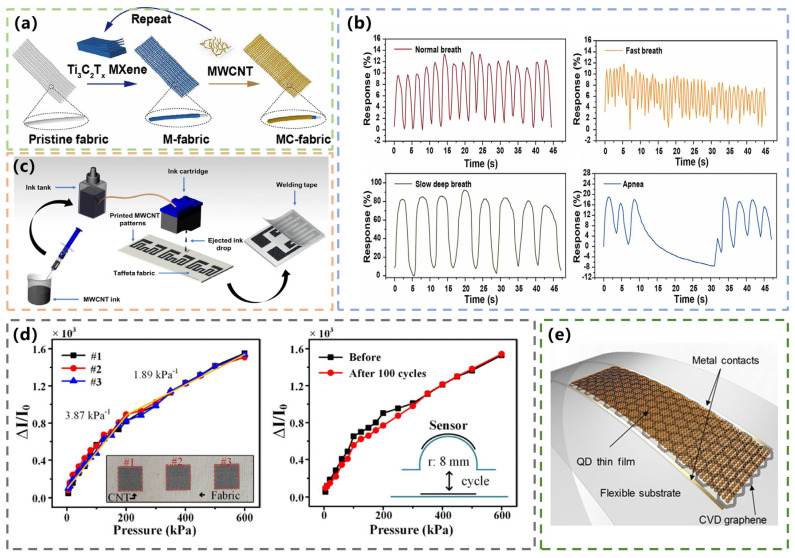
(**a**) Schematic process of the MC-Fabric humidity sensor. Reprinted with permission from Ref. [72]. Copyright 2022, ELSEVIER SCIENCE SA. (**b**) Respiration curves of four respiration patterns. Reprinted with permission from Ref. [72]. Copyright 2022, ELSEVIER SCIENCE SA. (**c**) The preparation process of the temperature sensor. Reprinted with permission from Ref. [73]. Copyright 2021, Springer. (**d**) The relationship between relative current change and pressure under different samples and bending. Reprinted with permission from Ref. [76]. Copyright 2022, IEEE. (**e**) Schematic of the assembly of graphene and QDs on a flexible substrate. Reprinted with permission from Ref. [79]. Copyright 2019, AMER ASSOC ADVANCEMENT SCIENCE.

Breathing can provide some information for the diagnosis of lung disease. The relative humidity of exhaled air is less affected by environmental factors such as temperature and movement. Therefore, the development of flexible humidity sensors for real-time continuous respiration monitoring is one of the most popular research topics. In 2022, Xing et al. reported a humidity sensor made of textile coated with MXene and MWCNT for real-time respiration monitoring. The researchers used alternate-drip coating to form MXene and MWCNT layers over a superfine fiber fabric (MC-Fabric) (Figure 7a). After MWCNT layers were inserted, the distance between MXene molecule layers became wider, which was conducive to the adsorption of more water molecules [71]. The response rate of the sensor was 265% at 90% relative humidity. The humidity response of the MC-Fabric sensor varied by less than 3% under both folded deformation and torsional deformation. The MC-Fabric could be integrated into a facemask to accurately identify various human breathing patterns. As shown in Figure 7b, one subject was tested for four representative breathing patterns including normal breathing, rapid breathing, slow deep breathing, and apnea. The breathing curve was visible, and different breathing patterns were clearly distinguished. The experimental results showed that the MC-Fabric sensor has good responsiveness and excellent repeatability, which provides a feasible method for real-time respiratory monitoring based on electronic textiles [72].

### 3.4. Carbon-Based Textile Sensors for Body Temperature Monitoring

Body temperature is often the simplest and most straightforward indicator of a person’s health. Many diseases are accompanied by changes in body temperature. Researchers in the field of flexible sensors have been working to achieve long-term continuous temperature measurements. The application of carbon-based materials in textile sensors for body temperature monitoring has also been studied. In 2021, Arman et al. developed a CNT-based temperature textile sensor. The conductive CNT-based ink was prepared by inkjet printing and deposited on Taffeta fabric. The surface was coated with a translucent PU film as a protective layer (Figure 7c). The sensor showed a negative temperature coefficient characteristic with which the resistivity of the sensor decreases with the increase in temperature. In addition, it can be used to measure human body temperature in real-time conditions [73].

### 3.5. Carbon-Based Tactile Textile Sensors

As an important way of perception, touch can make people respond differently to external mechanical stimuli. The research on tactile sensors is important, and carbon-based materials are also used in the production of tactile sensors. Bae et al. used inkjet printing technology to prepare a highly sensitive wearable CNT-based tactile sensor in 2022. It is difficult to form high-resolution patterns on textiles with printing techniques such as screen printing [74] and dip coating [75], which are commonly used in the production of textile sensors. To solve this problem, they used inkjet printing technology. Uniform CNT-based patterns were formed on cotton textiles, achieving a variety of high spatial resolutions of up to 0.5 mm patterns. Figure 7d shows the relative changes in the current of the sensor under different pressures and bending. The sensitivity of the sensor did not change significantly, indicating the repeatability of the method and the high flexibility of the sensor [76].

### 3.6. Carbon-Based HR and SpO_2_ Sensors

HR and SpO_2_ are important parameters of human health, and it is very important to monitor them for a long time, which can be applied to monitor diseases and exercise conditions. HR and SpO_2_ are usually measured by photoplethysmography (PPG). PPG is favored by researchers for non-invasive physiological-signal monitoring through the optical monitoring of blood flow [77,78]. In 2019, Polat et al. made a GOD (graphene sensitized with semiconducting quantum dots) photodetector. Because of its transparent nature, graphene can be used to make wearable photoelectric sensors. It uses flexible wearable devices and integrates with pre-designed electronic components (Figure 7e). It can detect vital signs that need to be tracked continuously for a long time, and it can work under ambient light [79].

## 4. Discussion and Outlook

In the past decades, textile sensors have drawn considerable research and commercial interest because of their flexibility, breathability, and ease of daily wear and use. Some carbon-based materials, including graphene [24,25,32,44,45,55,56,57,58,76], CNT [34,49,59,60,61,65,66,67,69,70,73], and CB [36,49,63,64], have been widely used in textile sensor research. Textile sensors are made by combining conductive carbon-based materials with flexible polymer textile materials. Flexible textile materials, such as cotton [73], nylon [44,45,63], silk [70], PU [32,56,58,61], and PVDF [58], are used as substrates or the basic structures of textile sensors. Carbon-based materials and textile materials are made into various physiological-signal sensors with excellent performance by coating [56,57,59,64], dipping [44,45,49], inkjet printing [70,73], electrospinning [32,61], the nano-soldering method [65,66], etc. The development of carbon-based textile sensors for physiological-signal monitoring pursues better performance, including higher sensitivity, faster response speed, lower hysteresis, and better linearity. In addition, researchers also pursue a higher sense of user experiences, such as better air permeability and comfort, better durability against bending caused by human movement or machine washing, and hydrophobic and anti-fouling characteristics. Table 1 shows the comparison of different carbon-based textile sensors for physiological-signal monitoring.

This review systematically summarizes the latest research progress of textile sensors combined with advanced carbon-based materials and introduces the design methods, preparation processes, and performances of textile sensors for monitoring different physiological signals. It can help readers or researchers in related fields understand the application of advanced carbon-based materials in the field of textile sensors and the development of flexible sensors in wearable devices for physiological-signal monitoring. Although various physiological-signal textile sensors incorporating new carbon materials have been reported, they are still far from practical application. While there are already some mature smart sensing clothes on the market, there are drawbacks such as poor washability and high prices. With the emergence and use of new materials and processes, textile sensors will have more innovative developments. Based on recent work on carbon-based textile sensors for practical applications, our views on possible directions and existing problems of textile sensors are shared in the following paragraphs.

### 4.1. Smart Textile Sensing System for Physiological-Signal Monitoring

Most integrated circuits are currently built on printed circuit boards (PCB), and even if they can be bent or stretched, they are difficult to design as flexible, breathable, and durable as textiles [80,81]. In addition, most of the current research on textile sensors for physiological-signal monitoring is limited to monitoring only one or several signals, and there is a lack of reports on integrating various functions into one textile. Therefore, the integration of multiple sensors into a textile circuit is a possible research direction in the field of physiological-signal sensors in the future. Researchers are working on smart textile sensing systems that can be used to monitor human physiological signals. 

**Figure 8:**
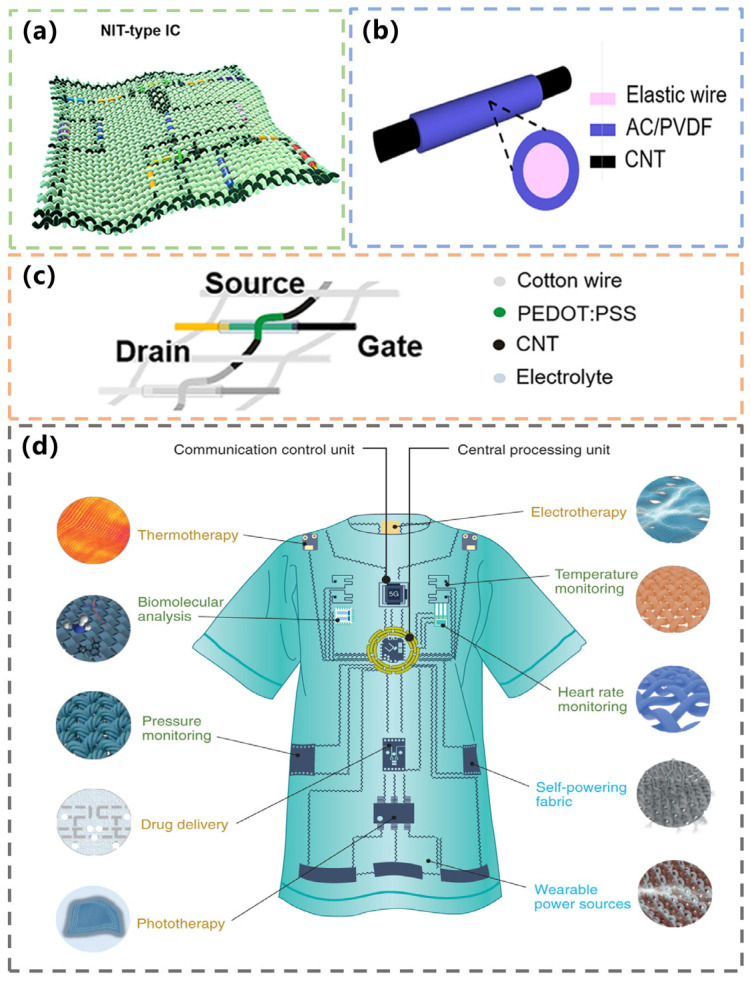
(**a**) Schematic of an NIT-type integrated circuit. Reprinted with permission from Ref. [82]. Copyright 2021, NATURE PORTFOLIO. (**b**) The structure of fabric-type strain sensors. Reprinted with permission from Ref. [82]. Copyright 2021, NATURE PORTFOLIO. (**c**) The structure of fabric-type transistors. Reprinted with permission from Ref. [82]. Copyright 2021, NATURE PORTFOLIO. (**d**) ATBANs on clothing. Reprinted with permission from Ref. [83] Copyright 2022, NATURE PORTFOLIO.

Researchers try to find new ways to combine integrated circuits with textiles. In 2021, Yang et al. reported a non-printed integrated-circuit textile (NIT) produced by a weaving method. The NIT contains a complete circuit structure that can be used for daily human health monitoring and treatment. In the NIT, all devices, including transistors, sensors, diodes, solar cells, etc., are assembled along polymer lines or at their intersections. Electrical connections between different devices can be achieved by making electrical contact at the crossing nodes between the CNT-coated parts of the warp and weft wires or by coating the conducting CNT parts along a wire. After weaving, all interwoven device nodes are encapsulated into a whole by PMMA. Figure 8a shows the basic structure of the NIT circuit. The green part is the cotton substrate, the black part is the interconnecting circuit, and the rest of the colored parts are the various fiber components. Almost all of the devices are fiber devices made by simple methods. In addition, the produced devices perform well. For example, in the production process of flexible stress sensors, a section of elastic wire coated with an activated carbon film (AC) is connected by a cotton wire coated with CNTs (Figure 8b). In the production process of transistors, a conductive PEDOT slice is deposited on a cotton thread, and each end of the PEDOT is a PSS layer that can be extracted by coaxially coating a layer of CNTs to form one or two conductive terminals on the cotton thread. Each device node of a fabric transistor has a PEDOT with a PSS/cotton wire electrode on the loom as the warp and another PEDOT with a PSS/cotton wire electrode entangled with a cotton wire at intervals as the weft (Figure 8c). By interweaving them together, they form a complete transistor. Many fabric-type transistors form a flexible circuit that performs logic functions [82].

Recently, Alberto et al. proposed a conceptual model of smart textiles called Autonomous Textile Body Area Networks (ATBANs) (Figure 8d). The smart fabric can provide a personalized medical solution, including diagnostic technology, a treatment platform, power supply technology, communication relay, and computing capabilities. Various flexible sensors and circuits are integrated into clothing. Body movement and physiological signals such as heart rate and body temperature are monitored in real-time, and timely medical intervention is achieved through the central processing unit and Internet of Things technology [83].

### 4.2. Interconnection of Textile Sensors

The interconnection between hard electronic device circuits and soft textile sensors in practical applications remains a noteworthy challenge. The interconnection requires it to be electrically and mechanically robust, while also being able to withstand the stretching and bending caused by human movement. In 2019, Seoane et al. interlinked conductive soft textile and hard electronic instrument components with fabrics with elastic and conductive CNT-based pastes. This method can guarantee the textile circuit’s flexibility while ensuring the monitored signal’s quality [84]. 

### 4.3. Problems of Textile Sensors in Practical Application

Most research on textile sensors only considers water resistance and does not consider the influence of other environmental factors on sensor performance. For example, there are few types of research on textile sensors in a high-temperature environment. In 2022, Peng et al. proposed a highly temperature-resistant and bendable textile sensor based on MWCNT, which can operate in the temperature range of 30–300 degrees Celsius [85]. In the future, high-temperature-resistant textile sensors have potential applications in aerospace, fire protection, etc. Therefore, the application stability of textile sensors in different or even harsh environments is a problem worth future consideration. Most of the current textile sensors can achieve high sensitivity, and some of them can maintain high sensitivity and have good tensile properties at the same time, but there is still a lot of research value and space on how to better achieve the balance between high sensitivity and high tensile properties. At present, textile-based sensors usually require additional layering before they can be manufactured directly on the fabric substrate. Flexible substrates, such as PDMS or PU, are also needed to protect the structure. This is because the surface of textiles is usually rough, which can result in weaker adhesion. Comfort may be affected in practical applications [86]. Carbon-based materials are widely used in flexible sensors because of their high electrical conductivity, thermal conductivity, and flexibility, but the low cost and large-scale production of high-performance carbon-based materials remain a challenge. Therefore, effective strategies such as functional materials and hybridization must be optimized to obtain materials suitable for wearable sensors [18]. Therefore, further research is needed to integrate wearable sensors into everyday clothing and provide long-term reliable physiological-signal monitoring.

## 5. Conclusions

Facing the need for wearable flexible sensors in physiological-signal monitoring, many advanced carbon-based material textile sensors are being studied. This review introduces the recent developments of textile sensors based on carbon-based materials for physiological-signal monitoring applications. The properties of carbon-based materials, the design structure, the basic principle, and the performance of the textile sensors are analyzed and discussed in detail, and a possible development direction of the future textile sensors is discussed. With the continuous development of textile sensors, it is believed that intelligent textile sensors with improved performance, higher comfort, greater stability, and better suitability for long-term daily health monitoring will be developed.

## Figures and Tables

**Table 1:** Comparison of the carbon-based textile sensors for physiological-signal monitoring mentioned above.

Physiological Signals	Carbon-Based Materials	Textile	Manufacturing Method	Advantages
ECG	rGO [44,45]	Nylon	Dipping	Cost-effective
	rGO, CB [32]	PU	Electrospinning	Good skin contact
	CNTs, CB [49]	Nonwoven	Dipping	Anti-fouling
Human motions [56]	Graphene	PU	Dip-coating	Sensing yarn, high sensitivity
Motion detection of figures [57]	GNPs	Lycra	Spin-coating	Excellent strain-resistance
Pulse, motions [58]	Graphene fibers	PVDF, PU	/	High GF, fast response time
Motion detection of figures [59]	CNTs	PVA yarn	Coating	Excellent stability
Human motions [60]	CNTs	Nylon–polyester- –spandex fabrics	Electrophoretic deposition	High comfortability
Human motions [61]	CNTs	PU	Electrospinning	High-pressure sensitivity, broad sensing range
Pulse [63]	CB	Nylon	/	High sensor repeatability
Human motions, biosignals [64]	CB	Fabric	Two-dimensional triaxial-braided weaving, dip coating	good stability and durability, environment-friendly
Human motions, pulse [65]	rGO, CNTs	Nonwoven	Nano-soldering	High sensitivity
Human motions [66]	rGO, CNTs	Nonwoven	Nano-soldering	High sensitivity, good washability and durability
Motion detection of figures [67]	GNSs, MWCNTs	Cloth	Screen-printing	Cost-effective, good stability
Respiration [69]	MWCNTs	Fabric	/	Improved humidity response and favorable response stability
Body temperature [70]	MWCNTs	Taffeta fabric	Inkjet printing	Negative temperature coefficient characteristic
Tactile perception [73]	CNTs	Cotton	Inkjet printing	High sensitivity, wide pressure- sensing range
HR and SpO_2_ [76]	Graphene	/	/	Long-term monitoring

## Data Availability

Not applicable.
